# Polymorphisms in BMP2/BMP4, with estimates of mean lung dose, predict radiation pneumonitis among patients receiving definitive radiotherapy for non-small cell lung cancer

**DOI:** 10.18632/oncotarget.17904

**Published:** 2017-05-17

**Authors:** Ju Yang, Ting Xu, Daniel R. Gomez, Xianglin Yuan, Quynh-Nhu Nguyen, Melenda Jeter, Yipeng Song, Stephen Hahn, Zhongxing Liao

**Affiliations:** ^1^ Department of Radiation Oncology, The University of Texas MD Anderson Cancer Center, Houston, TX, 77030, USA; ^2^ The Comprehensive Cancer Center of Drum Tower Hospital, Medical School of Nanjing University and Clinical Cancer Institute of Nanjing University, Nanjing, 210008, China; ^3^ Department of Oncology, Tongji Hospital, Huazhong University of Science and Technology, Wuhan, Hubei, 430030, China; ^4^ Department of Radiation Oncology, Yuhuangding Hospital, Shandong, 264000, China

**Keywords:** NSCLC, BMP2, radiation pneumonitis, polymorphism

## Abstract

Single nucleotide polymorphisms (SNPs) in *TGFβ1* can predict the risk of radiation pneumonitis (RP) in patients with non-small cell lung cancer (NSCLC) after definitive radiotherapy. Here we investigated whether SNPs in TGFβ superfamily members *BMP2* and *BMP4* are associated with RP in such patients. In total, we retrospectively analyzed 663 patients given ≥ 60 Gy for NSCLC. We randomly assigned 323 patients to the training cohort and 340 patients to the validation cohort. Potentially functional and tagging SNPs of *BMP2* (rs170986, rs1979855, rs1980499, rs235768, rs3178250) and *BMP4* (rs17563, rs4898820, rs762642) were genotyped. The median of mean lung dose (MLD) was 17.9 Gy (range, 0.15–32.74 Gy). Higher MLD was strongly associated with increased risk of grade ≥ 2 RP (hazard ratio [HR]=2.191, 95% confidence interval [CI] = 1.680–2.856, *P* < 0.001) and grade ≥ 3 RP (HR = 4.253, 95% CI = 2.493–7.257, *P* < 0.001). In multivariate analyses, *BMP2* rs235768 AT/TT was associated with higher risk of grade ≥ 2 RP (HR = 1.866, 95% CI = 1.221–2.820, *P* = 0.004 vs. AA) both in training cohort and validation cohort. Similar results were observed for *BMP2* rs1980499. *BMP2* rs3178250 CT/TT was associated with lower risk of grade ≥ 3 RP (HR = 0.406, 95% CI = 0.175–0.942, *P* = 0.036 vs. CC) in the pooled analysis. Adding the rs235768 and rs1980499 SNPs to a model comprising age, performance status, and MLD raised the Harrell's *C* for predicting grade ≥ 2 RP from 0.6117 to 0.6235 (*P* = 0.0105). SNPs in *BMP2* can predict grade ≥ 2 or 3 RP after radiotherapy for NSCLC and improve the predictive power of MLD model. Validation is underway through an ongoing prospective trial.

## INTRODUCTION

Lung cancer is the leading cause of cancer death worldwide, and non-small cell lung cancer (NSCLC) accounts for 85% of all lung cancer cases [1]. Approximately 70% of patients with NSCLC have locally advanced disease or distant metastases at diagnosis [2]. Radiotherapy is an important treatment modality for patients with locally advanced disease, particularly those with medical conditions that make them ineligible for surgery. However, radiation-induced lung toxicity, including radiation pneumonitis (RP) and subsequent lung fibrosis, limits the therapeutic ratio and can also complicate quality of life for sur*vivo*rs. Dosimetric variables such as mean lung dose (MLD) and V20 (the volume of lung exposed to radiation doses of 20 Gy or more) have been identified as being associated with RP [3, 4]. However, some patients will still develop RP even when these variables are under the thresholds established for them, implicating the patients’ genetic makeup in their response to radiotherapy.

At the cellular and tissue levels, RP is characterized by injury to type I and type II alveolar cells, endothelium, and fibroblasts, followed by infiltration of inflammatory cells, proliferation of type II alveolar cells and fibroblasts, progression through the epithelial-to-mesenchymal transition (EMT), remodeling of extracellular matrix, and deposition of collagen [5, 6]. The TGFβ superfamily includes multifunctional cytokines that have different isoforms and highly specific functions, e.g. wound healing, extracellular matrix remodeling, and EMT [7]. Patients who develop RP also show increases in levels of the cytokine TGFβ1 during radiotherapy [8]. We previously found that single nucleotide polymorphisms (SNPs) in *TGFβ1* could predict the risk of RP among patients receiving radiotherapy as definitive treatment for NSCLC [9]. A subgroup of the TGFβ superfamily, bone morphogenetic proteins (BMPs), is thought to influence inflammatory processes through their chemotactic effects on fibroblasts, myocytes, and inflammatory cells. In addition to the definite role of TGFβ1 in inducing EMT, BMP2 and BMP 4 are the best studied for EMT among the 20 different human BMPs. Interestingly, *BMP2* and *BMP4* have opposing functions: *BMP2* exerts pro-inflammatory effects in endothelial activation [10], whereas *BMP4* has anti-inflammatory effects in airway injury [11]. Previous study found that BMP2 was decreased and BMP4 was increased in idiopathic lung fibrosis[12]. Moreover, the ratio between BMPs and TGFβ1 correlates strongly with the EMT: increased TGFβ1 expression, decreased *BMP2* expression, and increased *BMP4* expression are all associated with induction of the EMT [12].

To the best of our knowledge, no studies have investigated potential associations between SNPs in *BMP2* and *BMP4* and the incidence of RP, and no studies have incorporated *BMP* SNPs into existing models based on MLD for predicting the risk of RP (grade ≥ 2 or 3) in patients after definitive radiotherapy for NSCLC. To address these gaps, we selected 8 potentially functional and tagging SNPs in *BMP2* (rs170986, rs1979855, rs1980499, rs235768, and rs3178250) and in *BMP4* (rs17563, rs4898820, and rs762642). Our hypothesis was that these SNPs in *BMP2* and *BMP4* are associated with incidence of RP in such patients and that incorporating these SNPs into an existing predictive model based on MLD could more accurately predict the risk of RP after definitive radiotherapy for NSCLC.

## RESULTS

### Patient characteristics

The same database was used as our previous study[13]. Characteristics of the study population are shown in Table 1. For the total population, the median age of the patients was 66 years (range 35–88 years), and most (488 [73.2%]) had stage III NSCLC. The median gross tumor volume (GTV) was 94.8 cm^3^ (range 1.5–1271.5 cm^3^), the median radiation dose was 69 Gy (range 60–87.5 Gy), and the median MLD was 17.9 Gy (range 0.15–32.741 Gy). Radiation was delivered as proton beam therapy to 139 patients (20.8%), as intensity-modulated (photon) radiotherapy to 331 patients (49.6%), and as 3-dimensional conformal radiotherapy to 174 patients (26.1%). In addition, 247 patients (36.3%) received induction chemotherapy and 560 patients (84.0%) received concurrent chemotherapy.

**Table 1:** Patient characteristics

Characteristic	Training cohort (*n* = 323)	Validation cohort (*n* = 340)	*P* value	Pooled analysis (*n* = 663)
**Age**				
< 66 (median)	160	171	0.877	331
≥ 66	163	169		332
**Sex**				
Male	184	178	0.243	362
Female	139	162		301
**Race**				
White	281	287	0.376	568
Other	42	53		95
**Disease Stage**				
I–IIIA	139	157	0.81	296
IIIB, IV, recurrence	160	172		333
**Tumor Histology**				
SCC	116	112	0.462	228
Non-SCC	207	228		435
**Karnofsky Performance**				
< 80	43	58	0.195	101
≥ 80	280	282		562
**Induction Chemotherapy**				
No	211	206	0.228	417
Yes	112	134		246
**Smoking Status**				
Never	19	36	0.034	55
Former/Current	298	298		596
**Total Radiation Dose, Gy**				
< 69.03 (median)	144	184	0.010	328
≥ 69.03	178	151		329
**Gross Tumor Volume, cm^3^**				
< 95.2 (median)	146	160	0.418	306
≥ 95.2	156	148		304
**Mean Lung Dose, Gy**				
< 17.9 (median)	162	157	0.579	319
≥ 17.9	154	165		319
**Radiation Modality**				
Photon (X-ray)	248	262	0.633	510
Proton	71	68		139

*P values of Pearson chi-square tests.

We randomized 323 patients to the training cohort and 340 patients to the validation cohort. The training cohort and the validation cohort matched well. These two cohorts showed no obvious distribution differences for age (*P* = 0.877), sex (*P* = 0.243), race (*P* = 0.376), disease stage (*P* = 0.81), tumor histology (*P* = 0.462), KPS (*P* = 0.195), induction chemotherapy (*P* = 0.228), GTV (*P* = 0.418), MLD (*P* = 0.579), and radiation modality (*P* = 0.633). Distributions of smoking status and total dose showed slight difference, however, as shown in Table 2, smoking status didn't contribute to the incidence of grade ≥ 2 RP (hazard ratio [HR] 0.825, 95% CI 0.532–1.279, *P* = 0.39) or grade ≥ 3 RP (HR 1.123, 95% CI 0.489–2.58, *P* = 0.784). No significant associations were shown between total dose and the risk of grade ≥2 RP (HR 0.799, 95% CI 0.622–1.026, *P* = 0.079) or grade ≥3 RP (HR 0.657, 95% CI 0.421–1.026, *P* = 0.065), either.

**Table 2:** Univariate and multivariate Cox regression analyses to identify clinical predictors of grade ≥ 2 and 3 radiation pneumonitis

Characteristic	grade ≥ 2 radiation pneumonitis						grade ≥3 radiation pneumonitis					
	Univariate Analysis			Multivariate Analysis*			Univariate Analysis			Multivariate Analysis*		
	HR	95% CI	*P*	HR	96% CI	*P*	HR	95% CI	*P*	HR	96% CI	*P*
Age (≥ 66 vs. < 66)	1.236	0.964–1.585	0.095	1.347	1.042–1.741	0.023	1.545	0.993–2.401	0.054	1.825	1.158–2.875	0.01
Sex (male vs. female)	1.005	0.784–1.290	0.966	NI			1.188	0.764–1.848	0.443	NI		
Race (black and other vs. white)	1.053	0.742–1.493	0.773	NI			1.048	0.568–1.934	0.881	NI		
Disease stage (IIIB, IV, recurrence vs. I–IIIA)	1.010	0.782–1.303	0.941	NI			0.779	0.499–1.217	0.273	NI		
Histology												
SCC & Other vs. Adenocarcinoma	0.954	0.743–1.224	0.711	NI			0.968	0.625–1.498	0.883	NI		
KPS (≥ 80 vs. < 80)	0.771	0.554–1.075	0.125	0.820	0.584–1.153	0.254	0.510	0.305–0.852	0.01	0.552	0.325–0.936	0.027
Induction chemotherapy (yes vs. no)	1.026	0.794–1.327	0.843	NI			1.246	0.802–1.938	0.328	NI		
Smoking status (current/former vs. never)	0.825	0.532–1.279	0.390	NI			1.123	0.489–2.580	0.784	NI		
Total dose (≥ 69.03 vs. < 69.03)	0.799	0.622–1.026	0.079	NI			0.657	0.421–1.026	0.065	NI		
Gross tumor volume (≥ 95.2 cm3 vs. < 95.2 cm3)	1.539	1.186–1.999	0.001	NI			2.76	1.678–4.539	< 0.001	NI		
Mean lung dose (≥ 17.9 Gy vs. < 17.9 Gy)	2.080	1.601–2.703	< 0.001	2.191	1.680–2.856	< 0.001	3.922	2.318–6.639	< 0.001	4.253	2.493–7.257	< 0.001
Radiation modality (proton vs. 3D–CRT + IMRT)	0.932	0.688–1.263	0.650	NI			0.65	0.359–1.176	0.154	0.766	0.419–1.4	0.386

*Characteristics with a *P* value of <0.05 in the univariate analysis were entered into the multivariate model in a stepwise fashion and were removed if at any point the *P* value was > 0.20.

*Abbreviations*: HR, hazard ratio; CI, confidence interval; NI, not included; KPS, Karnofsky Performance Status score; SCC, squamous cell carcinoma; 3D–CRT, 3–dimensional conformal (photon) radiation therapy; IMRT, intensity–modulated (photon) radiation therapy

### Clinical predictors of RP

The ability of characteristics shown in Table 1 to predict the incidence of RP of grade < 2 versus ≥ 2 or grade < 3 versus ≥ 3 were then evaluated in a stepwise fashion as follows. Variables found in univariate Cox regression analysis with *P* values of < 0.05 were entered into the multivariate analysis, and then removed if the *P value* was > 0.20. Significant associations were found in univariate analysis between the incidence of grade ≥ 2 RP and GTV (HR 1.539, 95% confidence interval [CI] 1.186–1.999, *P* = 0.001) or MLD (HR 2.080, 95% CI 1.601–2.703, *P* < 0.001) (Table 2). Because of the correlation between GTV and MLD, only MLD (the variable with larger HR and lower *P value*) was entered into the multivariate analysis. Multivariate analysis revealed that the risk of grade ≥ 2 RP was increased among patients who were ≥ 66 years old (HR 1.347, 95% CI 1.042–1.741, *P* = 0.023) and those whose MLD ≥ 17.9 Gy (HR 2.191, 95% CI 1.680–2.856, *P* < 0.001) (Table 2). Similar results were observed for the risk of grade ≥ 3 RP. Older patients or patients with higher MLD had greater risk of developing grade ≥ 3 RP. Besides, patients with higher KPS had the lower risk of grade ≥ 3 RP (HR 0.552, 95% CI 0.325–0.936, *P* = 0.027). In summary, these findings validated the already established role of MLD in the risk of RP.

### Associations between SNPs in *BMPs* and RP

**Figure 1:**
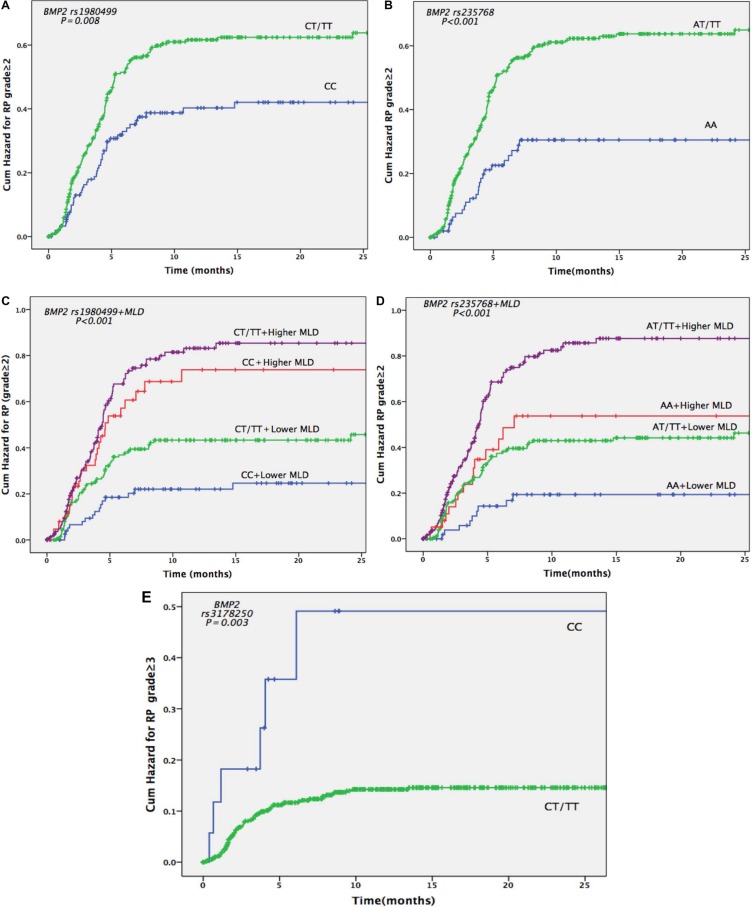
Cumulative probability of grade ≥ 2 or 3 radiation pneumonitis (RP) in 664 patients with non-small cell lung cancer according to *BMP2* genotypes (**A**) Effects of *BMP2* rs1980499 genotypes CT and TT versus CC on RP grade ≥ 2. (**B**) Effects of *BMP2* rs235768 genotypes AT and TT versus AA on RP grade ≥ 2. (**C**) Combined effects of *BMP2* rs1980499 genotypes and MLD on the risk of RP grade ≥ 2. (**D**) Combined effects of *BMP2* rs235768 genotypes and MLD on the risk of RP grade ≥ 2. (**E**) Effects of *BMP2* rs3178250 genotypes CT and TT versus CC on the risk of RP grade ≥ 3. The cut-off value for higher and lower MLD is 17.9 Gy (the median dose of MLD).

Next, we evaluated whether the *BMP* SNPs we genotyped (distribution shown in Supplementary Table 2) were associated with risks of grade ≥ 2 or grade ≥ 3 RP. The univariate analysis showed that *BMP2* rs235768 AT/TT genotypes were strongly associated with increased risk of grade ≥ 2 RP in the training cohort (HR 2.455, 95% CI 1.243–4.847, *P* = 0.010), validation cohort (HR 1.92, 95% CI 1.118–3.298, *P* = 0.018), and the pooled analysis (HR 2.090, 95% CI 1.370–3.187, *P* = 0.001) (Table 3). Similar results were also found in the multivariate analysis (training cohort: HR 2.186, 95% CI 1.103–4.333, *P* = 0.025; validation cohort: HR 1.884, 95% CI 1.093–3.246, *P* = 0.023; the pooled analysis: HR 1.866, 95% CI 1.221–2.820, *P* = 0.004) (Table 4; Figure 1B). For *BMP2* rs1980499, the univariate analysis of the training cohort suggested that genotypes CT/TT were associated with increased risk of grade ≥ 2 RP (HR 1.648, 95% CI 1.028–2.642, *P* = 0.038), while these results were not validated by the validation cohort (HR 1.461, 95% CI 0.946–2.256, *P* = 0.088). However, the pooled analysis still showed significant associations (HR 1.532, 95% CI 1.113–2.108, *P* = 0.009) (Table 3). The multivariate analysis in the pooled cohort also showed that *BMP2* rs1980499 genotypes CT/TT were associated with increased risk of grade ≥ 2 RP (HR 1.403, 95% CI 1.014–1.941, *P* = 0.041) (Table 4; Figure 1A).

**Table 3:** Univariate analysis of associations between single-nucleotide polymorphisms (SNPs) and grade ≥ 2 radiation pneumonitis

SNPs	Training Cohort			Validation Cohort			Pooled analysis		
	HR	95% CI	*P*	HR	95% CI	*P*	HR	95% CI	*P*
***BMP2***									
**rs170986 (CA/CC vs. AA)**	0.442	0.18–1.082	0.074	2.073	0.659–6.523	0.212	1.038	0.513–2.101	0.917
**rs1979855 (AG/AA vs. GG)**	0.522	0.165–1.651	0.268	1.853	0.458–7.495	0.387	1.049	0.432–2.545	0.916
**rs1980499 (CT/TT vs. CC)**	1.648	1.028–2.642	0.038	1.461	0.946–2.256	0.088	1.532	1.113–2.108	0.009
**rs235768 (AT/TT vs. AA)**	2.455	1.243–4.847	0.010	1.920	1.118–3.298	0.018	2.090	1.370–3.187	0.001
**rs3178250 (CT/TT vs. CC)**	0.364	0.177–0.748	0.006	0.954	0.303–3.003	0.936	0.529	0.288–0.969	0.039
***BMP4***									
**rs17563 (AG/GG vs. AA)**	0.844	0.563–1.266	0.413	1.280	0.843–1.942	0.246	1.047	0.783–1.400	0.756
**rs4898820 (GT/TT vs. GG)**	1.198	0.739–1.940	0.464	0.933	0.601–1.448	0.757	1.062	0.768–1.469	0.716
**rs762642 (AC/CC vs. AA)**	1.196	0.705–2.031	0.507	1.025	0.614–1.712	0.925	1.129	0.781–1.632	0.519

Abbreviation: HR, hazard ratio; CI, confidence interval.

**Table 4:** Multivariate analysis of associations between single-nucleotide polymorphisms (SNPs) and grade ≥ 2 radiation pneumonitis

SNPs	Training Cohort			Validation Cohort			Pooled analysis		
	HR	95% CI	*P*	HR	95% CI	*P*	HR	95% CI	*P*
***BMP2***									
**rs170986 (CA/CC vs. AA)**	0.442	0.179–1.093	0.077	1.666	0.522–5.319	0.389	0.921	0.451–1.882	0.822
**rs1979855 (AG/AA vs. GG)**	0.704	0.221–2.244	0.553	1.873	0.462–7.59	0.379	1.167	0.480–2.837	0.734
**rs1980499 (CT/TT vs. CC)**	1.557	0.957–2.533	0.075	1.457	0.937–2.265	0.095	1.403	1.014–1.941	0.041
**rs235768 (AT/TT vs. AA)**	2.186	1.103–4.333	0.025	1.884	1.093–3.246	0.023	1.866	1.221–2.820	0.004
**rs3178250 (CT/TT vs. CC)**	0.521	0.251–1.081	0.080	1.107	0.349–3.51	0.863	0.667	0.362–1.230	0.194
***BMP4***									
**rs17563 (AG/GG vs. AA)**	0.885	0.584–1.343	0.567	1.351	0.888–2.055	0.16	1.115	0.830–1.499	0.470
**rs4898820 (GT/TT vs. GG)**	1.347	0.811–2.236	0.25	0.909	0.584–1.415	0.673	1.115	0.800–1.555	0.521
**rs762642 (AC/CC vs. AA)**	1.303	0.753–2.254	0.345	1.006	0.601–1.685	0.982	1.156	0.794–1.682	0.449

Note: Age, Karnofsky Performance Status score, mean lung dose were used as covariates in multivariate analysis.

Abbreviation: HR, hazard ratio; CI, confidence interval.

The training cohort showed that *BMP2* rs3178250 genotypes CT/TT were obviously associated with the risk of grade ≥ 3 RP both in the univariate analysis (HR 0.272, 95% CI 0.108–0.687, *P* = 0.006) and multivariate analysis (HR 0.378, 95% CI 0.148–0.936, *P* = 0.041) (Table 5; Table 6). However, the validation cohort did not validate these results, but the pooled analysis still showed the significant associations in uni- (HR 0.303, 95% CI 0.132–0.698, *P* = 0.005) and multivariate analysis (HR 0.406, 95% CI 0.175–0.942, *P* = 0.036) (Table 6; Figure 1E).

**Table 5:** Univariate analysis of associations between single-nucleotide polymorphisms (SNPs) and grade ≥ 3 radiation pneumonitis

SNPs	Training Cohort			Validation Cohort			Pooled analysis		
BMP2	HR	95% CI	*P*	HR	95% CI	*P*	HR	95% CI	*P*
**rs170986 (CA/CC vs. AA)**	0.345	0.107–1.11	0.074	1.484	0.202–10.905	0.698	0.691	0.253–1.891	0.472
**rs1979855 (AG/AA vs. GG)**	0.870	0.120–6.316	0.890	0.680	0.092–5.005	0.705	0.818	0.201–3.335	0.779
**rs1980499 (CT/TT vs. CC)**	1.640	0.768–3.505	0.201	1.047	0.443–2.477	1.047	1.390	0.790–2.448	0.254
**rs235768 (AT/TT vs. AA)**	1.957	0.703–5.449	0.199	1.104	0.421–2.893	0.841	1.534	0.765–3.075	0.228
**rs3178250 (CT/TT vs. CC)**	0.272	0.108–0.687	0.006	0.524	0.071–3.870	0.524	0.303	0.132–0.698	0.005
***BMP4***									
**rs17563 (AG/GG vs. AA)**	0.806	0.434–1.499	0.496	2.452	0.853–7.048	0.096	1.182	0.698–2.001	0.535
**rs4898820 (GT/TT vs. GG)**	1.761	0.748–4.148	0.195	0.926	0.377–2.276	0.926	0.332	1.357–2.514	0.332
**rs762642 (AC/CC vs. AA)**	2.176	0.780–6.068	0.137	1.073	0.373–3.084	0.896	1.614	0.775–3.360	0.201

Abbreviation: HR, hazard ratio; CI, confidence interval.

**Table 6:** Multivariate analysis of associations between single-nucleotide polymorphisms (SNPs) and grade ≥ 3 radiation pneumonitis

SNPs	Training Cohort			Validation Cohort			Pooled analysis		
*BMP2*	HR	95% CI	*P*	HR	95% CI	*P*	HR	95% CI	*P*
**rs170986 (CA/CC vs. AA)**	0.393	0.120–1.282	0.122	0.945	0.124–7.196	0.957	0.579	0.208–1.613	0.296
**rs1979855 (AG/AA vs. GG)**	1.157	0.158–8.486	0.886	0.665	0.089–4.953	0.690	0.959	0.234–3.933	0.954
**rs1980499 (CT/TT vs. CC)**	1.454	0.675–3.132	0.339	1.028	0.432–2.447	0.950	1.216	0.688–2.147	0.501
**rs235768 (AT/TT vs. AA)**	1.655	0.591–4.632	0.337	1.027	0.390–2.704	0.956	1.307	0.650–2.626	0.452
rs3178250 (CT/TT vs. CC)	0.378	0.148–0.936	0.041	0.701	0.093–5.254	0.729	0.406	0.175–0.942	0.036
***BMP4***									
**rs17563 (AG/GG vs. AA)**	0.809	0.430–1.520	0.509	2.791	0.968–8.050	0.057	1.275	0.751–2.168	0.369
**rs4898820 (GT/TT vs. GG)**	1.763	0.746–4.168	0.196	0.957	0.386–2.370	0.924	1.414	0.761–2.627	0.273
**rs762642 (AC/CC vs. AA)**	2.139	0.764–5.987	0.148	1.080	0.374–3.121	0.887	1.638	0.785–3.418	0.188

Note: Age, Karnofsky Performance Status score, mean lung dose were used as covariates in multivariate analysis.

Abbreviation: HR, hazard ratio; CI, confidence interval.

### Incorporating SNPs into an clinical model

**Figure 2:**
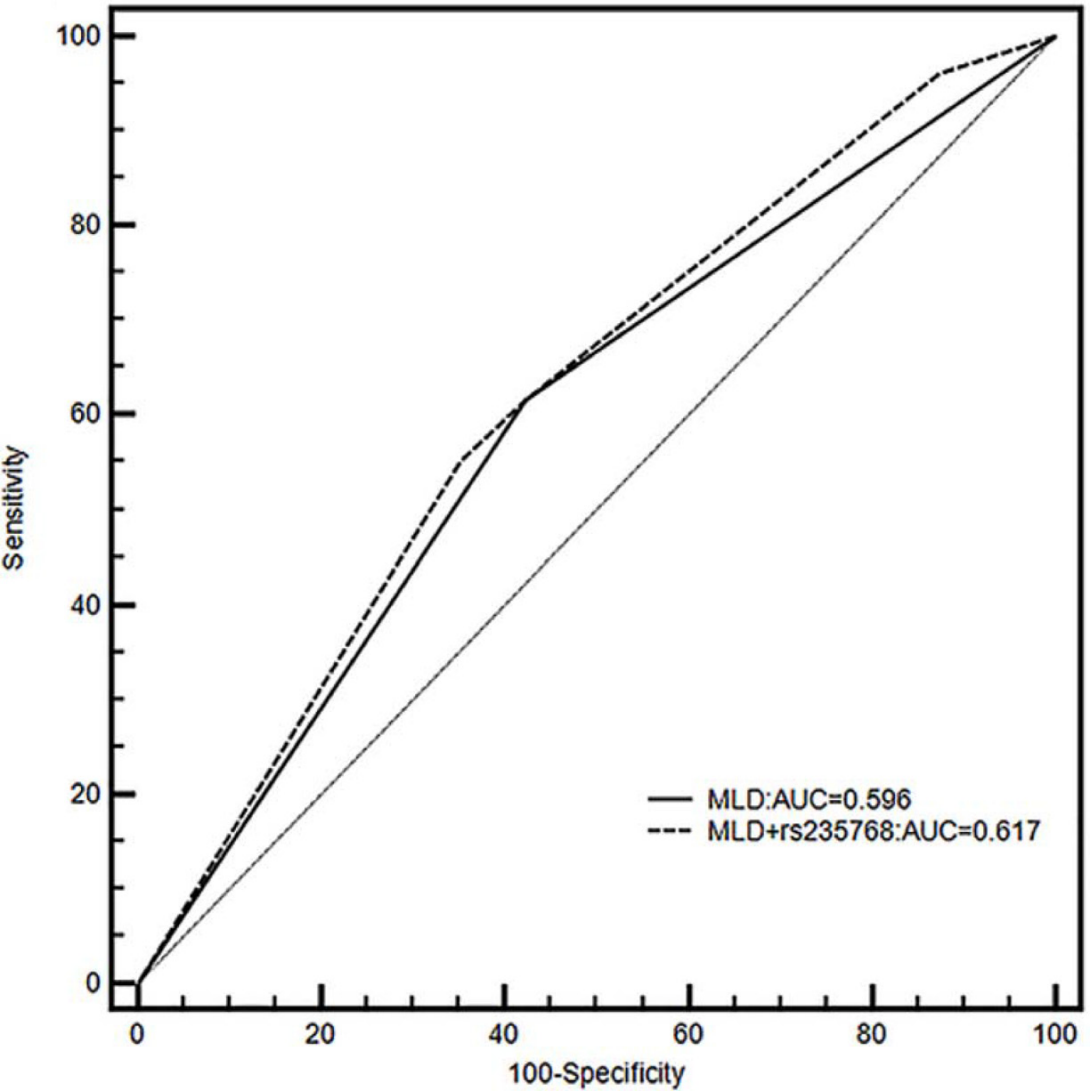
Receiver operating characteristics shown for models including only MLD (black line) or both MLD and rs235768 (dashed line) for grade ≥ 2 radiation pneumonitis (RP) with corresponding areas under curves (AUCs)

As shown in Figure 1C and 1D, the risk of developing RP ≥2 RP was highest in patients with *BMP2* rs1980499 gentoypes CT/TT and higher MLD or *BMP2* rs235768 AT/TT genotypes and higher MLD. Finally, we investigated if the two grade ≥ 2 RP associated *BMP2* SNPs (rs235768 and rs1980499) could improve the prediction of the clinical model consisted of age, KPS, and MLD [4]. To do so, we calculated Harrell's *C* to compare the predictive power of the models with or without the SNPs. The Harrell's *C* of the model without the SNPs was 0.6117, and it significantly increased to 0.6235 after the SNPs had been added (*P* = 0.0105). Receiver operating characteristics curve was also used to validate our results. As shown in Figure 2, a model containing MLD and rs235768 exhibited an AUC of 0.617, which was greater than model with MLD alone (AUC = 0.596). The difference between areas was also significant (95% CI: 0.00737–0.0346, *P* = 0.0025).

## DISCUSSION

To the best of our knowledge, this is the first study to investigate potential associations between SNPs in *BMPs* and the risk of RP. We found that *BMP2* SNPs rs235768 and rs1980499 were associated with risk of grade ≥ 2 RP; and rs3178250 was associated with the risk of grade ≥ 3 RP in patients with NSCLC after definitive radiotherapy. However, no association was found between *BMP4* SNPs and RP. Furthermore, we validated that higher MLD was a risk factor for RP and incorporating the SNPs (rs235768 and rs1980499) into an existing MLD-based model could more effectively predict the risk of RP.

Our findings also make sense from a biological standpoint. Like the general inflammation process, RP is a severe acute inflammatory response to radiation, followed by tissue repair and pulmonary fibrosis [14]. *BMP2* has complicated roles in this process, having been found to increase both during airway inflammation [15] and after exposure to pro-inflammatory stimuli such as TNFα. Early in the inflammatory process, increased expression of *BMP2* could have pro-inflammatory effects by inducing endothelial dysfunction, oxidative stress, and endothelial activation [10]. In the consequent tissue repair process, *BMP2* could have a novel role as a fibrosis-antagonizing cytokine through inhibiting TGFβ1 signaling [16, 17]. Therefore aberrant expression of *BMP2* could aggravate inflammation, induce more severe toxicity, or attenuate normal pulmonary tissue repair during radiation-induced lung injury. *BMP2* rs235768 is a non-synonymous mutation located in the coding area and may affect the protein product. Indeed, one study revealed that *BMP2* rs235768 could contribute to higher expression of *BMP2 in vitro* [18]. These mechanisms could explain our finding that the BMP2 rs235768 AT/TT genotypes could increase the risk of RP. *BMP2* rs1980499 is located within the transcriptional factor binding sites of *BMP2*. It may bind with either positive or inhibitory transcriptional factors and result in aberrant expression of *BMP2*.

One case-control study showing *BMP4* rs17563 increased the expression of *BMP4* mRNA and associated with the risk of cutaneous melanoma [19]. In this study we didn't find association between *BMP4* rs17563 and RP in NSCLC patients and couldn't be able to validate the *BMP4* expression.

The ability to safely escalate radiation doses has long been considered important for patients with cancer given its established dose-response relationship [20, 21]; however, results from the Radiation Therapy Oncology Group trial 0617 showed that a dose of 74 Gy (given in 2-Gy fractions with concurrent chemotherapy) not only was no better than 60 Gy with concurrent chemotherapy for patients with stage III NSCLC but also may have been harmful [22]. This result may have reflected limitations on the assessment of radiation-related toxicity and its contribution to treatment-related death. Therefore, it is crucial to identify patients who will benefit from higher radiation dose as well as those patients who are at high risk of developing radiation-related toxicity. Our inclusion of SNPs related to RP with dosimetric parameters related to RP represents a helpful tool for identifying those patients who are at increased risk of RP and thus may be less suitable for high-dose radiation. From the perspective of clinical practice, testing SNPs is inexpensive and relatively less invasive than analysis of tumor samples, as SNPs can be tested directly in peripheral blood samples, which expands the numbers of patients who can undergo this testing.

A strength of our study was the relatively large number of patients analyzed (663); indeed, this may be the largest study reported to date on associations between SNPs and RP in patients with NSCLC [23–25]. We further tested the power of adding SNPs to the established clinical RP predictive model to investigate the predictive role of SNPs in the aspect of clinical practice.

However, this study also had some limitations. First, because it is a single institution retrospective study, these findings await for validation by other institutions and other patient populations. Second, we did not investigate the mechanisms underlying our positive results. Third, only potentially functional and tagging SNPs were studied, rather than all of the SNPs in the entire gene.

In conclusion, we found that SNPs in *BMP2* (rs235768, rs1980499, and rs3178250) can predict grade ≥ 2 or 3 RP after definitive radiotherapy for NSCLC, and including these SNPs in an existing model predicting RP risk based on age, performance status, and MLD could improve the predictive power.

## MATERIALS AND METHODS

### Study population

This retrospective analysis was approved by the institutional review board of The University of Texas MD Anderson Cancer Center, and we complied with all applicable Health Insurance Portability and Accountability Act regulations. We searched an institutional database to identify patients who had received definitive radiotherapy for NSCLC at MD Anderson from 1999 through 2014 who had (1) histologically confirmed NSCLC; (2) received a total radiation dose of ≥ 60 Gy [or ≥ 60 Gy (RBE) for proton therapy]; (3) available computed tomography (CT) or positron emission tomography (PET) scans obtained within 1 year after completing radiotherapy, to be used for detecting and scoring RP; and (4) available archived blood samples for genotyping. Patients who had received stereotactic ablative radiotherapy were excluded. A total of 663 patients met these criteria and were the subjects of this analysis.

RP was assessed at each follow-up visit after the completion of radiotherapy and graded according to the National Cancer Institute's Common Terminology Criteria for Adverse Events version 3.0. Follow-up visits took place within the first 1–3 months after radiotherapy and then every 3 months thereafter for 2 years; those visits included interval history and physical examinations and imaging studies.

### SNP selection and genotyping methods

**Figure 3:**
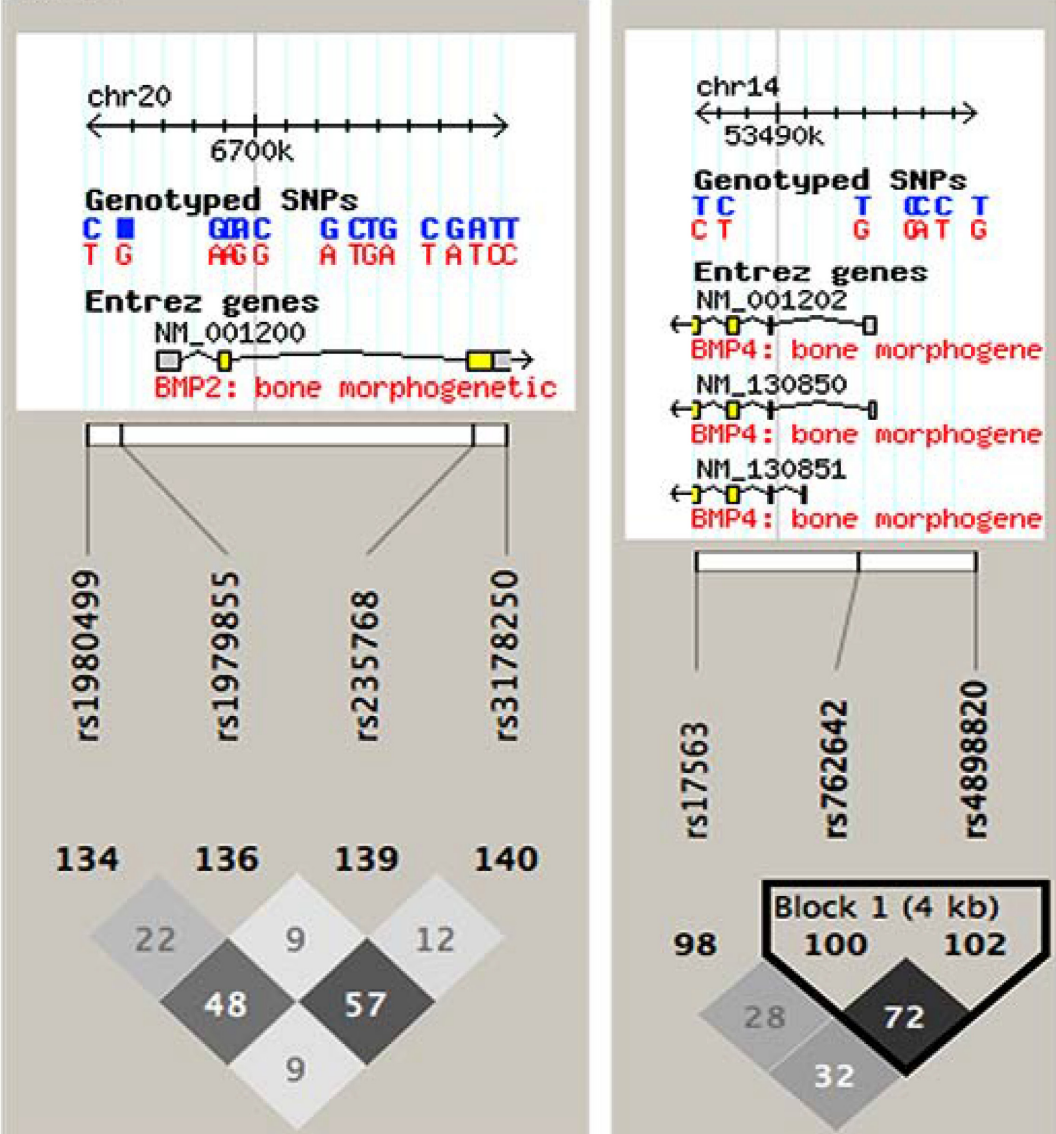
Linkage disequilibrium map for the single nucleotide polymorphisms studied The numbers shown in the grid represent R^2^ × 100 values. R^2^ of our studied SNPs ranged from 0.09 to 0.72, all less than 0.80, indicating that our studies SNPs were not in high linkage disequilibrium.

Potentially functional and tagging SNPs were selected by using https://snpinfo.niehs.nih.gov/snpinfo/snpfunc.html. Predicted SNP functions are shown in Supplementary Table 1. The inclusion criteria were: (1) a minor allele frequency of > 5% among whites; (2) located in a transcription factor binding site, a microRNA binding site, or a non-synonymous mutation in the coding area; and (3) an R^2^ value for linkage disequilibrium between each SNP of < 0.8. Linkage disequilibrium was calculated with Haploview (https://www.broadinstitute.org/scientific-community/science/programs/medical-and-population-genetics/haploview/haploview) (Figure 3; numbers in the grid are R^2 ×^ 100).

Genomic DNA was extracted from the buffy coat fraction of blood samples using a blood DNA mini kit. The genomic DNA was genotyping by Taqman real-time polymerase chain reaction. The extract of genomic DNA and genotyping methods were described in our previous study [13]. For all genotypes, the assay success rate was > 95%, and concordance of repeated sample testing was 100%.

### Statistical analyses

Chi-square tests were used to test for potential differences in distributions. Potential associations between RP risk and genotypes were assessed with a Cox proportional hazard model, with consideration of time to event. Characteristics with *P* values < 0.05 in the univariate Cox analysis were entered into the multivariate analysis; characteristics with a *P value* of > 0.20 in the multivariate analysis were then removed. Kaplan-Meier analysis was used to assess the effect of different genotypes on the cumulative probability of RP. All of these analyses were done with SPSS 22. Harrell's *C* was calculated to determine if adding SNPs that were found to be significant in the multivariate analyses could improve the ability of a model comprising age, performance status, and MLD to predict the risk of RP. All tests were 2-sided and differences were considered significant at *P* < 0.05.

## SUPPLEMENTARY TABLES


