# Supplementary material for: Polymorphisms in BMP2/BMP4, with estimates of mean lung dose, predict radiation pneumonitis among patients receiving definitive radiotherapy for non-small cell lung cancer
Source: Oncotarget. 2017 May 17;8(26):43080–90. doi: 10.18632/oncotarget.17904 (PMC5522129; doi:10.18632/oncotarget.17904)
Supplement: Supplementary file 1 [file oncotarget-08-43080-s001.pdf]

Polymorphisms in BMP2/BMP4, with estimates of mean lung dose, predict radiation pneumonitis among patients receiving definitive radiotherapy for non-small cell lung cancer

Supplementary Materials

Supplementary Table 1: Function prediction of tagging SNPs of BMP2 and BMP4

SNPs	Chromosome	Allele	TFBS	miRNA	nsSNP
Rs170986	20	A/C		Y	
Rs1979855	20	G/A	Y		
Rs1980499	20	C/T	Y		
Rs235768	20	A/T			Y
Rs3178250	20	C/T		Y	
Rs17563	14	G/A			Y
Rs4898820	14	G/T	Y		
Rs762642	14	C/A	Y		

Abbreviations: TFBS, transcriptional factor binding site; nsSNP, non-synonymous single nucleotide polymorphism.

Supplementary Table 2: Genotypes distribution of studied SNPs

	No. of patients	
**SNPs**	**RP grade < 2**	**RP grade ≥ 2**
**Rs170986**		
**AA**	16	8
**AC**	116	74
**CC**	268	156
**Rs1980499**		
**CC**	288	179
**CT**	102	53
**TT**	10	5
**Rs235768**		
**AA**	81	24
**AT**	156	103
**TT**	161	113
**Rs3178250**		
**CC**	8	11
**CT**	131	68
**TT**	261	164
**Rs1979855**		
**AA**	288	179
**AG**	102	53
**GG**	10	5
**Rs17563**		
**AA**	105	61
**AG**	171	109
**GG**	122	72
**Rs4898820**		
**GG**	82	45
**GT**	185	116
**TT**	126	77
**Rs762642**		
**AA**	165	93
**AC**	162	107
**CC**	63	33
